# *Kaempferia parviflora* extract and its methoxyflavones as potential anti-Alzheimer assessing in vitro, integrated computational approach, and in vivo impact on behaviour in scopolamine-induced amnesic mice

**DOI:** 10.1371/journal.pone.0316888

**Published:** 2025-03-10

**Authors:** Pitchayakarn Takomthong, Pornthip Waiwut, Chavi Yenjai, Jinda Wangboonskul, Kusawadee Plekratoke, Puguh Novi Arsito, Carlo Ballatore, Chantana Boonyarat

**Affiliations:** 1 Faculty of Pharmaceutical Sciences, Khon Kaen University, Khon Kaen, Thailand; 2 Faculty of Pharmaceutical Sciences, Ubon Ratchathani University, Ubon Ratchathani, Thailand; 3 Faculty of Sciences, Khon Kaen University, Khon Kaen, Thailand; 4 Faculty of Pharmacy, Thammasat University, Bangkok, Thailand; 5 School of Pharmacy, Faculty of Medicine and Health Sciences, Universitas Muhammadiyah Yogyakarta, Yogyakarta, Indonesia; 6 Skaggs School of Pharmacy and Pharmaceutical Sciences, University of California, San Diego, San Diego, California, United States of America; Mahidol University, THAILAND

## Abstract

Alzheimer’s disease (AD), a growing global challenge, lacks effective preventive and therapeutic strategies. This study explored the promising potential of the *Kaempferia parviflora* (KP) and its methoxyflavones (MFs) against the disease. We evaluated KP extract and its five MFs for antioxidant capacity, cholinesterase inhibition (AChE, and BChE), amyloid plaque (Aβ) reduction, neuroprotection, and memory improvement in a mouse model. HPLC quantified the five MFs in KP extract, with 5,7-dimethoxyflavone (F1) being the most abundant. 5,7,4′-Trimethoxyflavone (F3) and 5-hydroxy-3,7-dimethoxyflavone (F4) exhibited the strongest AChE and BChE inhibitory activities, respectively. MFs hindered Aβ_1-42_ aggregation and destabilized fibrils, with F3 showing the potent anti-aggregation and the strongest fibril destabilization. They also protected SH-SY5Y cells from Aβ_1-42_-induced damage. Notably, F3 combined anti-cholinesterase and anti-Aβ activities, suggesting its potential as a multi-target agent. KP extract ameliorated scopolamine-induced memory deficits in mice, suggesting its potential for cognitive improvement. These findings revealed that KP can be a promising candidate for herbal medicine development against AD. Its multi-target MFs offered a unique advantage by targeting multiple AD pathways. KP may have a great potential to modify the disease and overcome the challenge of drug development as cognitive enhancing herbal medicine.

## Introduction

The aging global population has increased significantly in recent years. The world’s population of people aged 60 years and older is 1.4 billion and is projected to double in 2050, reaching 2.1 billion [1]. Several diseases are strongly correlated with aging, including cancers, cardiovascular diseases, neurodegenerative diseases and so on. Alzheimer’s disease or AD is one of the chronic neurodegenerative diseases which is ranked as the seventh most cause of death in US and it is the most frequent cause of dementia [2]. The characteristics of AD are the accumulation of extracellular amyloid beta (Aβ) aggregates and intracellular neurofibrillary tangles (NFTs). Additionally, other hypotheses based on the pathophysiological changes during AD progression include the depletion of acetylcholine (ACh) neurotransmitter and oxidative stress. It is well established that one of major mechanisms leading to neuronal death in AD is the excessive Aβ accumulation [3,4]. Given the current AD therapies and medications, they remain largely ineffective, expensive, and may have adverse effects [5]. Now on, current therapies for AD primarily focus on symptomatic relief and targeting specific aspects of the disease, such as cholinesterase inhibitors and NMDA receptor antagonists. However, these treatments often provide limited efficacy and do not address the full spectrum of AD pathology [6]. Given the diverse mechanisms involved in AD, there is a growing consensus that a multi-action therapeutic approach could be more effective in treating the disease. Multi-action therapies aim to simultaneously target several pathological processes, potentially leading to better clinical outcomes [7].

In recent years, herbal medicines have been gaining popularity as complementary or supplementary treatments for various illnesses. The use of herbal medicine for AD is a topic of ongoing research and debate. While there is currently no known cure for AD, herbal remedies are sometimes used as a complementary approach to conventional treatments to manage symptoms and improve overall well-being. Some herbs have been studied for their potential effects on AD, including Ginkgo biloba [8–10]. This herb has been traditionally used in Chinese medicine for centuries. It has been studied for its potential effects on cognitive function and memory, and some studies have suggested that it may have a modest benefit in improving AD symptoms [8,10,11]. Additionally, some active components in herbal plants possess pharmacological effects related to AD [12]. For instance, curcumin from turmeric has been studied for its potential anti-inflammatory and antioxidant effects. Some studies have suggested that curcumin may have a protective effect on the brain and may help to improve cognitive function in people with AD [12,13]. Huperzine A is a compound derived from a Chinese herb called *huperzia serrata*. It has been studied for its potential effects on cognitive function and memory, and some studies have suggested that it may have a modest benefit in improving symptoms of AD [12,14].

*Kaempferia parviflora* Wall. ex Baker (KP), a member of the Zingiberaceae family, has emerged as a promising candidate for therapeutic applications. Traditionally used for health promotion and cognitive enhancement [15], KP exhibits a diverse range of pharmacological activities, including anti-inflammatory, anti-allergic, antioxidant, and neuroprotective properties [16,17]. Previous studies have demonstrated KP’s potential in addressing cognitive impairments and neurodegenerative diseases. For instance, KP extract has been shown to counteract cognitive impairments in rats [16], elevate key neurotransmitters involved in emotion, learning, and memory (such as dopamine and serotonin) [18] and suppress Aβ-induced neuroinflammation and oxidative stress in stem cells [17], Moreover, the primary chemical constituents of KP extract, methoxyflavones, possess activities relevant to AD [19,20]. Given its multifaceted properties, KP extract is a promising candidate to address the limitations of current AD therapies and provide more effective treatment options for patients.

**Fig 1:**
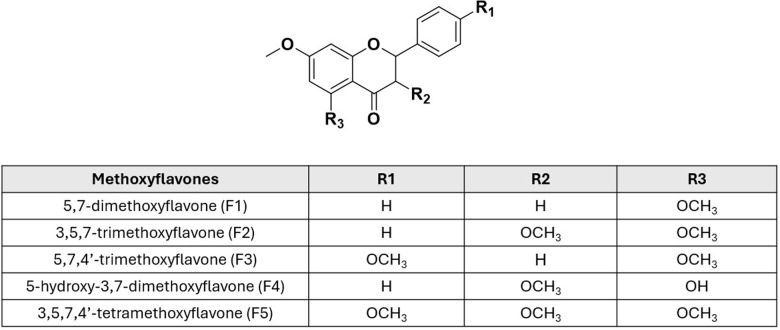
Chemical structures of five methoxyflavones.

This study delves deeper into KP’s potential as a multi-target herbal medicine against AD by investigating its five main active flavonoids (5,7-dimethoxyflavone (F1), 3,5,7-trimethoxyflavone (F2), 5,7,4′-trimethoxyflavone (F3), 5-hydroxy-3,7-dimethoxyflavone (F4), and 3,5,7,4′-tetramethoxyflavone (F5)) alongside the crude ethanol extract (Fig 1). We assessed their anti-Alzheimer’s activities, including their ability to scavenge free radicals, inhibit cholinesterase (AChE and BChE), reduce Aβ aggregation and protect neurons from Aβ toxicity. To gain deeper insights, we further explored the molecular interactions between MFs and the key AD targets. The structure-activity relationship analysis elucidated the precise mechanisms underlying KP’s multi-faceted neuroprotective effects. Additionally, KP’s memory-enhancing effects were investigated in a scopolamine-induced mouse model to validate its potential for cognitive improvement. Therefore, this study contributed a significant understanding of KP’s multi-target therapeutic potential against AD. By validating its efficacy in modulating key AD pathways and enhancing cognitive function, this research paves the way for KP’s development as a safe and effective herbal medicine for preventing and managing neurodegenerative diseases. Importantly, this study underscored the promising role of multi-target herbal medicine in addressing the complex challenges posed by AD and other age-related neurodegenerative diseases.

## Methods

### Preparation and characterization of the KP extract

The fresh rhizome of KP, a plant found in Loei province, Thailand, were collected (3 kg). Dr. Prathan Luecha identified and vouchered the specimen (CY 4303) at the Herbarium of Khon Kaen University’s Faculty of Pharmaceutical Science. To investigate its potential bioactive properties, the KP was extracted with 95% ethanol at 1:5 (w/v) ratio for 7 days at room temperature. KP extract were concentrated by filtering, and then evaporating under reduced pressure using a rotary evaporator. The concentrated extract was freeze-dried for complete dryness. The KP extract was stored in airtight containers at 4˚C. Five methoxyflavones (F1 to F5) were extracted from KP rhizome, and these compounds were previously isolated and structurally characterized [19,20].

Analysis of the KP extract and five MFs was performed using an Agilent 1260 HPLC system (Agilent Technologies). Separation on a Hypersil BDS C18 column (4.6 x 100 mm, 3 µm) used a gradient elution method with 0.5% acetic acid in water (A) and acetonitrile (B) as the mobile phase at a flow rate of 1 mL/min. The column temperature was maintained at 25°C, and 10 µL injections were analyzed. The total runtime of the method was 40 min, with the concentration gradient varying as follows: 65% A and 35% B at 0 min, 20% A and 80% B at 14 min, 20% A and 80% B 25 min, 0% A and 100% B at 30 min, 65% A and 35% B at 31 min, and 65% A and 35% B at 40 min. The chromatograms were detected at wavelength 264 nm. Quantification of MFs was based on peak area (AUC) using a pre-established calibration curve.

### In vitro studies

#### Determination of phenolic contents.

The widely method for determining the total phenolic contents is Folin-Ciocalteu method [21]. Briefly, 10 µL of KP extract or gallic acid was mixed with 75 µL of 10% (w/v) Folin–Ciocalteu reagent. Then, 7.5% of Na_2_CO_3_ was added after 5 min. The UV absorbance was determined at wavelength 700 nm after incubation for 2 hours. The total phenolic content of the KP extract was quantified as milligrams of gallic acid equivalents per gram of dry extract (mg GAE/g).

#### Determination of flavonoid contents.

Twenty µL of KP extract solution or quercetin was mixed with 15 µL of 10% (w/v) AlCl_3_ solution in ethanol, 20 µL (1 M) sodium acetate and 160 µL of distilled water. The absorbance is measured at 450 nm after mixture incubation for 15 min at room temperature. The total flavonoid content of the KP extract was quantified as milligrams of quercetin equivalents per gram of dry extract (mg QE/g) [21].

#### DPPH and ABTS radical scavenging activity.

DPPH and ABTS methods were used to measure the antioxidant activity of KP extract and methoxyflavones [21]. Briefly, for the DPPH assay, 100 µL of 0.2 mM DPPH solution was mixed with the KP extract or MFs at room temperature. The mixtures were kept in the dark for 30 minutes and then the absorbance was measured at 550 nm. In case of ABTS assay, ABTS radical cation (ABTS • + ) was prepared from a mixture of the ABTS stock solution (7 mM) and potassium persulfate (2.45 mM) in water. The mixture was then kept in the dark for 12-16 hours before use. Then, the ABTS • +  solution was diluted in ethanol until the absorbance reached 0.70 ± 0.02 at 700 nm. The KP extract or MFs were added to the ABTS • +  solution, incubated in the dark for 15 minutes and determined the absorbance.

Trolox was used as a positive control in both assays, while a mixture of ethanol and DPPH or ABTS • +  solution served as the negative control. The percentage inhibition of DPPH and ABTS radicals was calculated using the following equation:


$$DPPHandABTSinhibition\left(\%\right)=\frac{\left(A_{0}-A_{1}\right)}{A_{0}}\times 100$$

A_0_ is the absorbance of the control and A_1_ is the absorbance of the sample.

#### Cholinesterase inhibitory activity.

The modified Ellman’s method was used to determine the inhibitory activity of cholinesterase [22]. The solution of 25 µL of KP extract or MFs or positive controls (tacrine and donepezil), 25 µL of ATCI or BTCI, 125 µL of DTNB, and 50 µL of enzyme (AChE or BChE) was mixed and incubated and measured the absorbance at 405 nm every 30 seconds for 5 minutes. A negative control was the mixture solution without both cholinesterase.

#### Inhibition of amyloid beta (Aβ _1-42_ ) aggregation, destabilization, and kinetic studies.

Aβ_1-42_ was firstly dissolved in HFIP and aliquoted to 250 µM to obtain Aβ_1-42_ monomers. Then, the solvent was dried in hood using nitrogen, and reconstituted to working solution with 0.5 M phosphate buffer (pH 7.4) [23]. To determine the Aβ_1-42_ aggregation, the Aβ_1-42_ was mixed and incubated with KP extract, MFs, or curcumin for 48 hours at 37˚C. For Aβ_1-42_ destabilization, the Aβ_1-42_ monomers were preincubated at 37˚C for three days to generate aggregates, and then subsequently incubated with or without KP extract, MFs, or curcumin for 24 hours. Thioflavin T (ThT) is used to investigate the amyloid fibril formation and exhibit enhanced fluorescence when it binds to amyloid fibrils. ThT was used to monitor through the fluorescent changes and measured at wavelengths of 446 nm (excitation) and 490 nm (emission). The most potent MF and KP extract were recorded in their fluorescence intensity at 0 to 18 hours for kinetic assay.

#### Neuroprotective effect against amyloid beta (Aβ _1-42_ )-induced cell damage.

Neuroblastoma cells (SH-SY5Y) were maintained in humidified atmosphere at 37˚C with 5%CO_2_ in DMEM/F12 supplement with 10%FBS. Afterward, cells were treated for 6 days and replaced every 3 days with 1%FBS supplementation and 10 μM of retinoic acid [24]. Cells were seeded (100,000 cells per well) in 96-well microplates and cultured for 48 hours before treatment. To prepare aggregated Aβ_1-42_, lyophilized Aβ_1-42_ was reconstituted in sterile water and stored at –80 ˚C. Aliquots were diluted to a final concentration of 25 µM in serum-free medium and incubated at 37 ˚C for 72 hours to induce amyloid aggregation. For the assay, cells were pretreated with the KP extract or MFs at different concentrations for 2 hours. After removing unabsorbed test compounds, the cells were incubated with aggregated Aβ_1-42_ at a final concentration 25 µM for 24 hours to induce cell damage. Curcumin at a concentration of 10 µM served as the positive control. Cell viability was assessed using MTT assay. Absorbance at 570 nm was measured for both treated and untreated samples, with viability expressed as a percentage relative to the control.

### Computational studies

The 3D structure of AChE (PDB ID: 2CEK), BChE (PDB ID: 1P0I), Aβ_1-42_ monomer (PDB ID: 1IYT) and fibril (PDB ID: 2BEG) were downloaded from Protein Data Bank. The optimization of the 3D structure of MFs was used Chem3D 22.2.0 for energy minimization through the MM2 force field. Using Autodock 4.0, all waters and the original ligands were removed, hydrogens and charges were merged and added to the proteins, respectively. Afterwards, all templates were validated and docked with MFs. All grid sizes were set to cover the active site of protein with a spacing of 0.375 Å. During the docking, the Lamarckian genetic algorithm parameter was used to consider 200 conformations. All binding conformation were visualized and depicted with Discovery studio 2020.

### In vivo studies

#### Preparation of drugs.

Scopolamine was dissolved in NSS. Donepezil and crude extract were dissolved in corn oil. Animal doses were calculated according to mice body weight. The dose of scopolamine (1 mg/kg) were used to induce cognitive deficit, and the dose of donepezil (3 mg/kg) showed a good therapeutic effects in the prevention of scopolamine-induced memory impairment [21].

#### Ethical statements and humane endpoints.

The KKU ethical committee for animal experimentation reviewed and approved the experiment protocols (approval number: IACUC-KKU-30/66). All research staff received training in animal care and use according to Thai IACUC guidelines. To ensure humane treatment, a protocol for establishing humane endpoints was developed and followed daily. This protocol relied on health and welfare assessments of the laboratory animals. Animals were euthanized upon displaying signs of pain, suffering, or distressful behavior changes as identified by a mouse grimace scale. Three mice exhibited such signs and were humanely euthanized immediately following sedation with thiopental sodium. Otherwise, all animals were euthanized at the experimental endpoint (day 31) using cervical dislocation after sedation with thiopental sodium (120 mg/kg/BW, i.p.). No animal died before meeting criteria for euthanasia.

#### Acclimatization and classification of animals.

**Fig 2:**
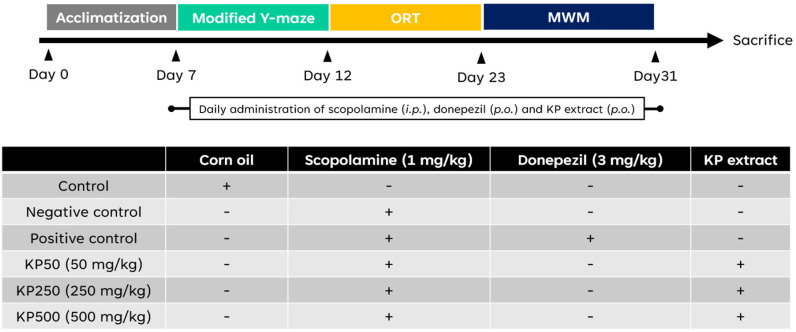
Experimental design and schedule.

Sixty male ICR mice in age 6 weeks (34-44 g) were supplied from Northeast Laboratory Animal Center, KKU. Before conducting the experiments, they were acclimatized for a week with a 12h light-dark cycle and had free access to their diet and water. Their body weight was recorded every day. Sixty mice were randomly divided into six groups and received different treatment as follows: (1) control group, which was given corn oil alone; (2) scopolamine group, which was injected with scopolamine alone via intraperitoneal injection (i.p.); (3) donepezil group, which was administered donepezil per oral (p.o.) and was injected with scopolamine; (4) KP50, which was administered KP extract (50 mg/kg) per oral and was injected with scopolamine; (5) KP250, which was administered KP (250 mg/kg) per oral and was injected with scopolamine; (6) KP500, which was administered KP (500 mg/kg) per oral and was injected with scopolamine. As shown in Fig 2, the treatment or vehicle was orally administered 1 hour prior to scopolamine injection. Except control group, amnesia was induced by intraperitoneally scopolamine injection 30 minutes before conducting experiments. Mice should be healthy for behavioral testing, and they were excluded if they remain immobile and do not respond to challenges. Investigators could not be blinded to the mouse groups due to the difference in code colors. However, the investigators who assessed the mouse’s behavior were blinded to the mouse code for providing unbiased data. The parameters related to behavioral changes were assessed including the number of arm entries, the exploration time spent, and the time spent in a preference quadrant.

#### Locomotor activity test.

Locomotor activity was investigated in a black polyvinyl chamber Y maze. The size of the chamber is 40 cm long, 3 cm wide at the bottom and 10 cm wide at the top, and 12 cm high in each arm. Mice were first allowed to acclimate the chamber and freely moved in Y maze for 8 minutes. After acclimatization, the locomotor activity was measured for 1 hour following the treatment administration. The number of arm entries was recorded from the middle of the maze.

#### Modified Y-maze test.

A modified Y maze is different from a Y maze using in locomotor activity which it has a black partition which used to close one of three arms. In sample phase, mice were placed in the maze with one closed arm. To investigate the short-term memory, mice freely explored the other two arms for 5 minutes. Thirty minutes later, mice were placed with all opened-arms, and explored freely for 5 minutes in test phase for evaluating the short-term memory. After 24 hours, mice were placed again, and allowed to explore in all open arms for evaluating the long-term memory. To prevent bias from olfactory cues, 70% ethanol was used to clean all arms area and closed partition after each trial. When mice assessed each arm at least 10 cm, the number of entries was visually counted. The percentage of unfamiliar arm exploration was calculated using the following equation:


$$Unfamiliararmexploration\left(\%\right)=\frac{Numberofunfamiliararmentry}{Numberofallarmsentry}\times 100$$

#### Novel object recognition test.

The novel object recognition test was performed in an open field box (52 x 52 x 40 cm). Mice were expected to discriminate between the objects through the different shapes. There are two identical objects (familiar object; F) which are white cylinder shapes in acquisition phase, whereas the new object (N) is a white rectangular shape in the test phase. Prior to the day before the experiments, mice were placed near the side wall and freely explored the open field for 5 minutes in habituation phase. During the acquisition phase, two identical objects were placed in two corners at 10 cm from the sidewall. Mice were placed in the open field box and explored the identical objects for 5 minutes. All areas and objects were cleaned with 70%ethanol to eliminate the olfactory cues before starting each phase. Then, the one object was changed to the new one, and mice were put back to re-expose two different objects (familiar and new objects) in test phase for 5 minutes. After a delay of 24 hours, the test phase was re-evaluated for long-term memory assessment. The different time points in an experiment referred to the investigation of short and long-term memory. The exploration time spent was recorded using a stopwatch when mice approached objects within 3 cm. The discrimination index (DI) was calculated as follows:


$$Discriminationindex\left(DI\right)=\frac{\left(TN-TF\right)}{\left(TN+TF\right)}\times 100$$

TN refers to time spent with new object, whereas TF refers to time spent with familiar object.

#### Morris water maze test.

The Morris water maze consists of a large black circular pool filled with water (20 ± 2˚C, 30 cm deep) to cover a black platform which was hidden below the water surface. It was in the center of one quadrant of the pool. In the acquisition phase, the platform was set in a specific quadrant and mice were trained to find the platform. They were released into the water at each quadrant and freely swam for 60 seconds. If mice failed to find the platform, they were gently guided to the platform and allowed to stay on the platform for 10 seconds. Mice were released and trained continuously for 5 days. To ensure their learning memory, the platform was removed on day 6, and mice were released and allowed freely to swim for 60 seconds in probe test. The time spent in a preference quadrant was recorded and analyzed.

### Statistical analysis

All data are presented as mean ±  SD and mean ±  SEM for in vitro and vivo studies, respectively. Statistical significance of differences between means was established by ANOVA with Tukey’s post hoc tests. All statistical calculations were performed using SPSS (IBM SPSS Statistics v.27) with an accepted significance of * *p* < 0.05 and **p < 0.01 in all tests.

## Results

### Analysis of KP extract and MFs chemical profiles

**Fig 3:**
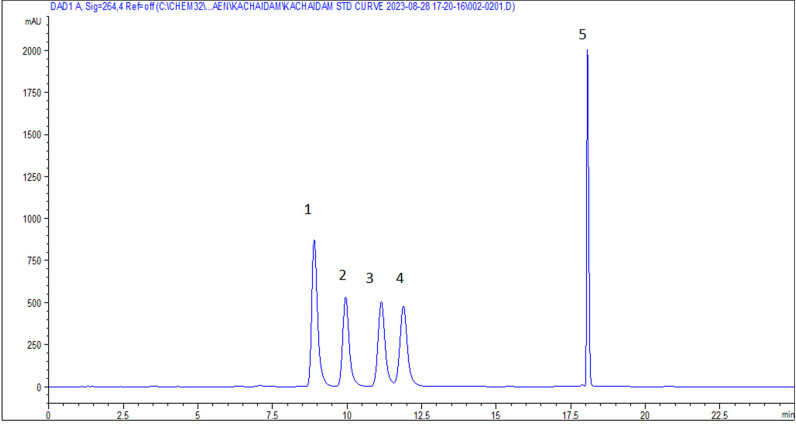
HPLC Chromatogram of five MF standards in mobile phase system of (A) 0.5% acetic acid in water and (B) acetonitrile at a flow rate of 1 mL/min. (Retention time; 1; F1 (RT 8.900); 2; F3 (RT 9.955); 3; F2 (RT 11.149); 4; F5 (RT 11.890); 5; F4 (RT 18.059)).

**Fig 4:**
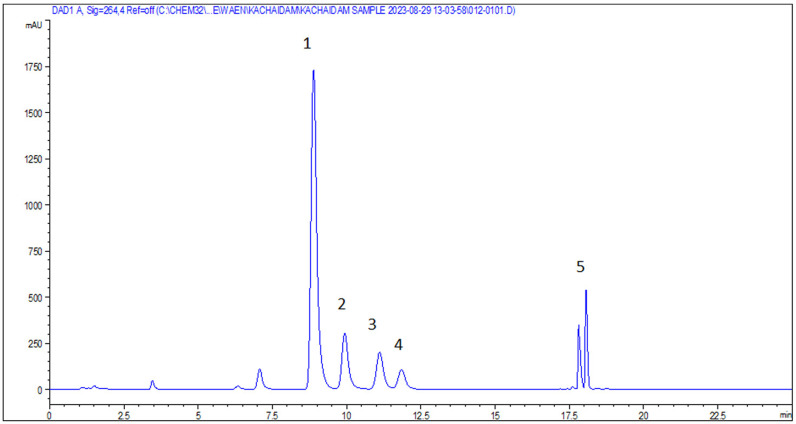
HPLC Chromatogram of KP extract (1.0 mg/ml) in mobile phase system of (A) 0.5% acetic acid in water and (B) acetonitrile at a flow rate of 1 mL/min. (Retention time; 1; F1 (RT 8.888); 2; F3 (RT 9.936); 3; F2 (RT 11.111); 4; F5 (RT 11.848); 5; F4 (RT 18.060)).

The crude yield of KP extract obtained using 95% v/v ethanol maceration was 6.74%. High-performance liquid chromatography (HPLC) was employed to identify the chemical constituents of the KP extract by comparing its chromatogram to five standard methoxyflavones (MFs). The retention times of the KP chromatogram peaks were matched with those of the standard MFs. The results confirmed the following order of MF content in the KP extract: F1 (33.88%w/w), F3 (10.04%w/w), F2 (7.30%w/w), F4 (6.00%w/w), and F5 (3.67%w/w). The structures of five MF standards are depicted in Fig 1. All peaks were identified as standard compounds based on their specific UV absorption at 264 nm (Figs 3 and 4).

### In vitro assessment of KP extract and its MFs on activities related to AD pathogenesis

#### Quantification of total phenolic and flavonoid contents in KP extract.

The determination of total phenolic content, commonly expressed as milligrams of gallic acid equivalents (GAE) per milligram of solid crude, provides a quantitative assessment of phenolic compounds within crude extract. Phenolic compounds exert their antioxidant effects through diverse mechanisms involving interactions with various types of free radicals such as hydrogen atom transfer, single electron transfer and so on [25]. The determination of total flavonoid content in the extract utilized a spectrophotometric method involving aluminum chloride. The flavonoid content was quantified and expressed as micrograms of quercetin equivalents (QE) per milligram of solid crude. Our results revealed that KP ethanolic extract presented a total phenolic content and total flavonoid content of 66.14 ± 1.66 mg GAE/g solid crude, and 57.18 ± 2.87 QE/g solid crude.

#### Assessment of antioxidant activity in KP extract and MFs using DPPH and ABTS assays.

The antioxidant activities of KP extract were tested using the ABTS and DPPH assays. The ABTS assay measures the ability of antioxidants to quench the ABTS radical cation, which is generated by the reaction of ABTS with potassium persulfate. The DPPH assay, on the other hand, involves the reduction of the DPPH radical, a stable free radical, by antioxidants. When an antioxidant is present, it donates an electron or a hydrogen atom to the radicals. Both assays are commonly used to evaluate the free radical scavenging activity of various compounds [26]. In our study, the KP extract exhibited antioxidant activity, with ABTS and DPPH IC_50_ values of 307 ± 3.41 µg/mL and 552 ± 2.89 µg/mL, respectively. Trolox was used as a standard reference. In the other hand, MFs showed no activity in either the DPPH or ABTS assays.

#### Cholinesterase inhibition.

Cholinergic neurons are widely distributed in the human brain and play a role in cognition. In neuron signaling, AChE is an essential enzyme in cholinergic synapses that could degrade ACh, terminate the neurotransmission on the postsynaptic membrane. Therefore, the inhibition of the AChE, the catabolic enzyme of ACh, can contribute to an increase in ACh brain level [27]. Although BChE is mainly expressed in serum, liver, and heart, it is also found in CNS, especially glial cells. Unlike AChE, it is less efficient in ACh hydrolysis. The essential role of BChE in the brain is to support ACh hydrolysis [28–30]. The inhibition of KP extract against ChE was determined by the Ellman’s method and showed the IC_50_ values against AChE and BChE of 100.61 ± 1.29 and 22.99 ± 1.15 µg/mL, respectively. Furthermore, five MF also showed the IC_50_ values against AChE and BChE ranged from 106.00 to 237.15 µM and 33.45 to 233.15 µM, respectively (Table 1).

**Table 1:** In vitro biological activities related to Alzheimer’s pathology (IC_50_) of MFs and KP extract. Data are represented as mean ±  SD (n =  3).

Test compounds	AChE inhibition	BChE inhibition	Inhibition of Aβ formation	Aβ destabilization	Antioxidant	
					DPPH	ABTS
**KP extract (µg/mL)**	100.61 ± 1.29	22.99 ± 1.15	198.93 ± 3.31	4.13 ± 0.12	552.36 ± 2.89	306.73 ± 3.41
**F1 (µM)**	181.30 ± 2.66^c^	92.19 ± 0.69^c^	105.16 ± 1.34^c^	75.93 ± 4.96^d^	>100	>100
**F2 (µM)**	237.15 ± 2.28^e^	233.15 ± 6.89^f^	87.13 ± 4.51^b^	83.86 ± 1.67^e^	>100	>100
**F3 (µM)**	106.00 ± 1.14^b^	151.98 ± 6.14^d^	91.40 ± 4.67^b^	39.47 ± 0.61^b^	>100	>100
**F4 (µM)**	217.57 ± 1.84^d^	33.45 ± 2.97^b^	97.10 ± 2.38^bc^	63.93 ± 1.83^d^	>100	>100
**F5 (µM)**	209.08 ± 0.82^d^	164.25 ± 5.13^e^	134.26 ± 5.08^d^	51.26 ± 3.13^c^	>100	>100
**Tacrine (µM)**	0.22 ± 0.00^a^	0.01 ± 0.00^a^	nd	nd	nd	nd
**Donepezil (µM)**	0.07 ± 0.00^a^	9.52 ± 0.36^a^	nd	nd	nd	nd
**Curcumin (µM)**	nd	nd	7.36 ± 0.34^a^	6.66 ± 0.37^a^	nd	nd
**Trolox (µM)**	nd	nd	nd	nd	24.63 ± 0.39	81.43 ± 4.67

a-f Means in a row with different superscript letter represent statistically significant differences (*p* <  0.05) nd: non-detectable.

#### Inhibition of Aβ _1-42_ aggregation and destabilization.

**Fig 5:**
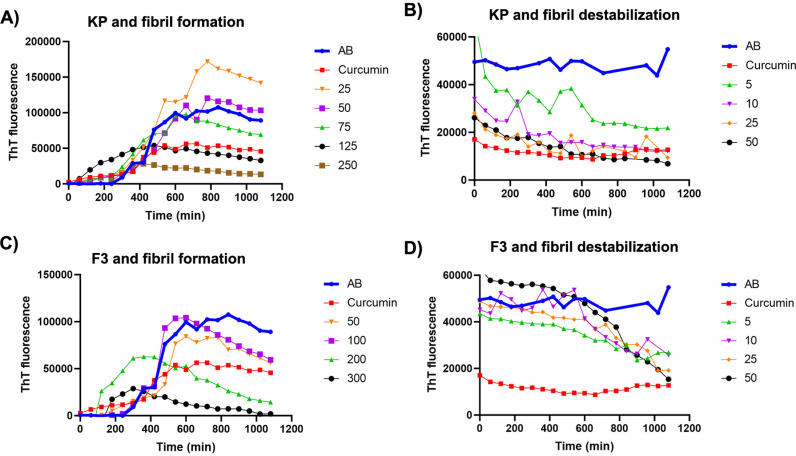
Effects of KP extract and F3 on Aβ_1-42_ fibril formation and destabilization of preformed fibrils. Inhibition of Aβ_1-42_ fibril formation by KP extract (A) and F3 (C) at various concentrations. Destabilization of preformed Aβ_1-42_ fibrils by KP extract (B) and F3 (D) at various concentrations. All are measured by ThT fluorescence assay. Curcumin (10 µM) was used as a positive control.

The formation of Aβ fibrils is a key pathological feature of AD, and there is considerable interest in understanding the mechanisms underlying this process to develop new therapeutic approaches. ThT is used to examine the fibril formation of Aβ species because it is known to bind only to the fibrillar forms of Aβ species [31,32]. Aβ_1-42_ monomers or fibrils (fAβ) were incubated in their presence and absence to test whether KP extract, and MFs could inhibit the formation of Aβ_1-42_ and promote fibril destabilization. The most potential MF and KP were chosen and investigated a time-course measurement of the fluorescent intensity of ThT bound to Aβ_1-42_ monomer or preformed Aβ_1-42_ fibrils. During the 18-hour time course of the experiment, there was a concentration-dependent relationship. The inhibitory effects of KP extract and MFs on Aβ_1-42_ fibrillization and destabilization were calculated as IC_50_ values and presented in Table 1. MFs exhibited IC_50_ values in the range of 87.13 to 134.26 µM. Moreover, MF interacted with the fibrils and destabilized fAβ with IC_50_ values ranging from 39.47 to 83.86 µM. Among five MFs, F3 possessed the ability to inhibit and destabilize Aβ_1-42_ fibrils compared to the other compounds. Therefore, the time-course measurements of Aβ_1–42_ fibrillization and destabilization in the presence and absence of the test compounds were investigated and represented in Fig 5C and 5D. We found that F3 inhibited Aβ_1–42_ fibrillization and destabilization in a dose-dependent manner. In the case of KP extract, it possessed both abilities to inhibit and destabilize Aβ_1-42_ with the IC_50_ of 198.93 ± 3.31 and 4.13 ± 0.12 µg/mL, respectively. Moreover, it was also studied its effects on Aβ_1–42_ fibrillization and destabilization, and the results revealed that KP extract exhibited a concentration-dependent in both activities (Fig 5A and 5B).

#### Neuroprotective effect against Aβ _1-42_ -induced cell damage.

**Fig 6:**
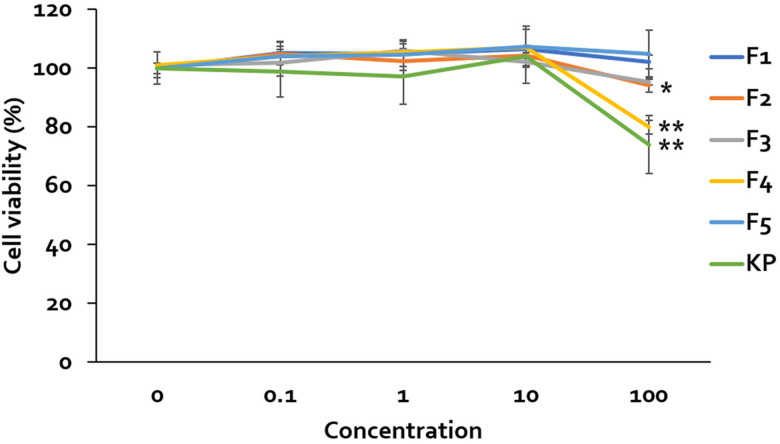
Cytotoxicity evaluation of MFs and KP extract. Cell viability of SH-SY5Y cells treated with various concentrations of MFs and KP extract, assessed by the MTT assay. Data are presented as mean ±  SD. Statistical significance is indicated as * p <  0.05, **p <  0.01 compared to the untreated control group.

**Fig 7:**
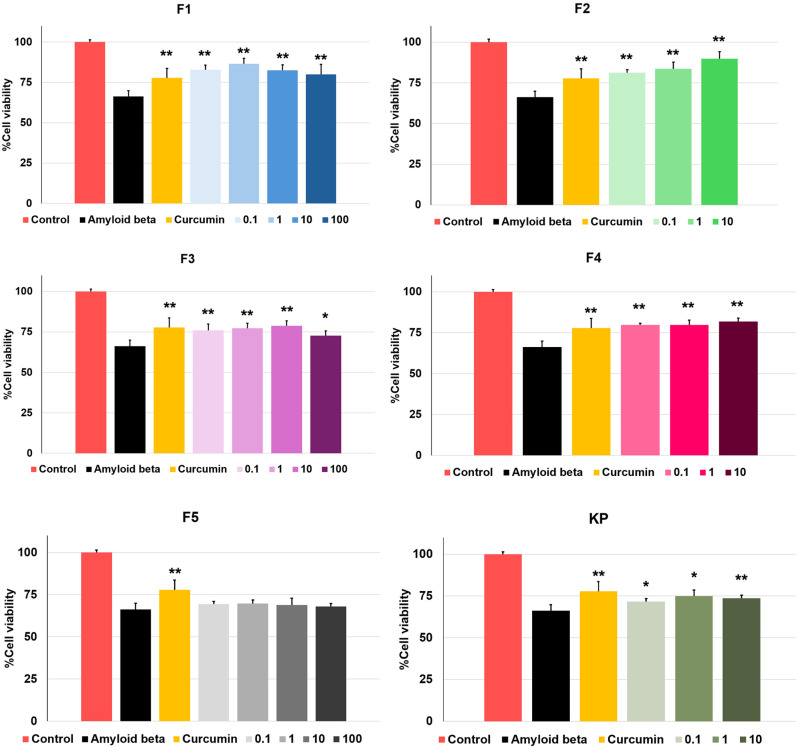
Neuroprotection evaluation against Aβ-induced cell damage. Cell viability of SH-SY5Y cells treated with 25 µM of aggregated Aβ peptides followed by various concentrations of MFs, and KP extract, assessed by the MTT assay. Data are represented as mean ±  SD. Statistical significance is indicated as * p <  0.05, **p <  0.01 compared to the Aβ-treated group. Curcumin at 10 µM (CUR) was used as a positive control.

The cell viability of the differentiated SH-SY5Y was determined by the MTT assay. MFs and KP extract were tested and considered to be safe, showing no toxicity within range of 0.1 to 100 µM. However, F2, F4 and KP extract at concentration 100 µM are toxic to cells (Fig 6). Therefore, these concentrations will not be used to evaluate the protective effect against Aβ_1-42_-induced cell damage. The differentiated SH-SY5Y cells have been treated with Aβ_1-42_ at concentration 25 µM for 24 h. It reduced cell survival to 66.24 ±  3.66% compared to control cells. To evaluate the neuroprotective effect of MFs and KP extract, they were pre-treated in the cells following Aβ_1-42_. The addition of MFs and KP extract restored cell survival, except all concentration of F5 (Fig 7).

### Computational techniques

#### Principle component analysis (PCA).

**Fig 8:**
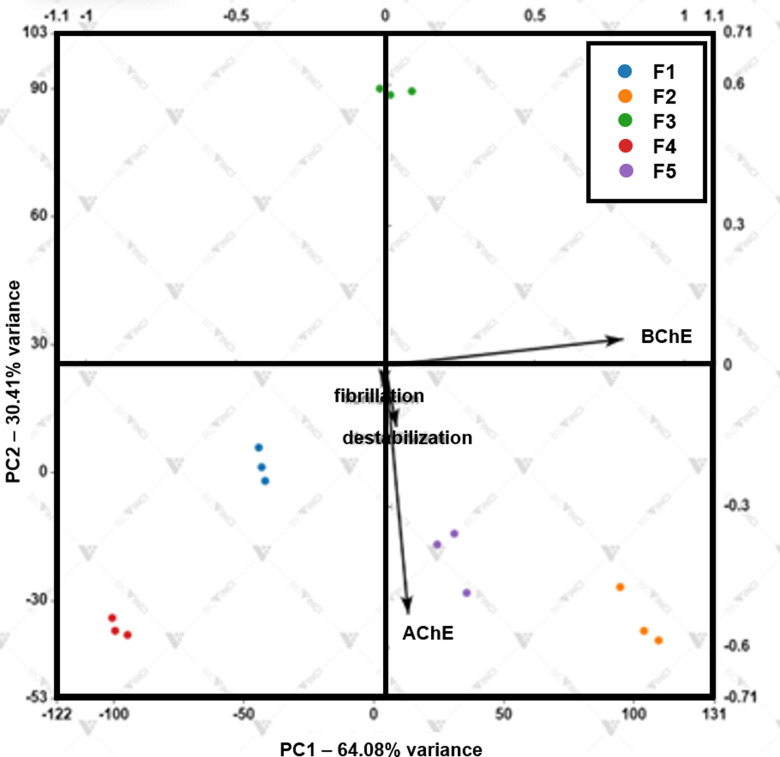
Principal Component Analysis (PCA) of IC_50_ values and anti-AD activities (anti-ChE, anti-Aβ aggregation, and Aβ destabilization) of MFs.

PCA is a versatile tool that aids in understanding and identifying the ley influential variables. Herein, it was used to explore the link between MFs and anti-AD activities using BioVinci (Bioturing, San Diego, CA, USA). PCA of MFs indicated that PC1 explained 64.08% of total variances. Cumulative contribution of PC1 and PC2 reached 94.49% which explained it can accurately represent the data. Each methoxyflavones were separated in the score plot. F1 and F4 are in the negative parts of both PC1 and PC2, while F3 is in the positive parts of both PC1 and PC2. F2 and F5 are groups along in the positive parts of PC1 and they are also in the negative parts of PC2. As a result, PC1 explained anti-BChE, whereas PC2 was found to be negatively associated with anti-AChE, anti-Aβ aggregation, and Aβ destabilization. F3 was well separated from the other methoxyflavones by being on the positive parts of PC2 due to its significantly higher anti-AChE, anti-Aβ aggregation, and Aβ destabilization. Thus, it can be stated that anti-AChE, anti-Aβ aggregation, and Aβ destabilization as three variables are useful in clustering F3 from the others separately (Fig 8).

#### Molecular docking and interaction analysis.

**Fig 9:**
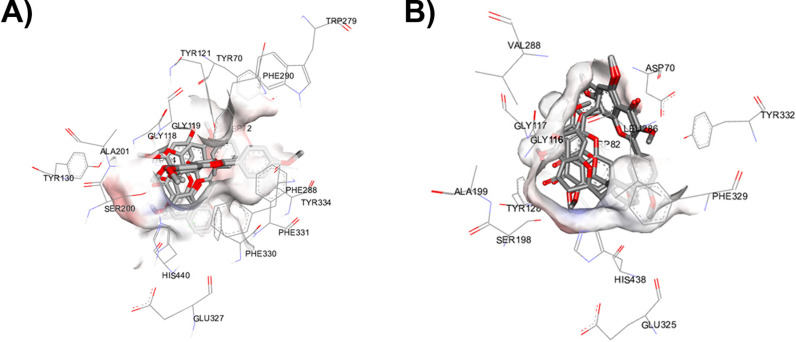
Predicted binding modes of MFs (Methoxyflavones) at the active sites of (A) acetylcholinesterase (AChE) and (B) butyrylcholinesterase (BChE), as determined by molecular docking simulations.

**Fig 10:**
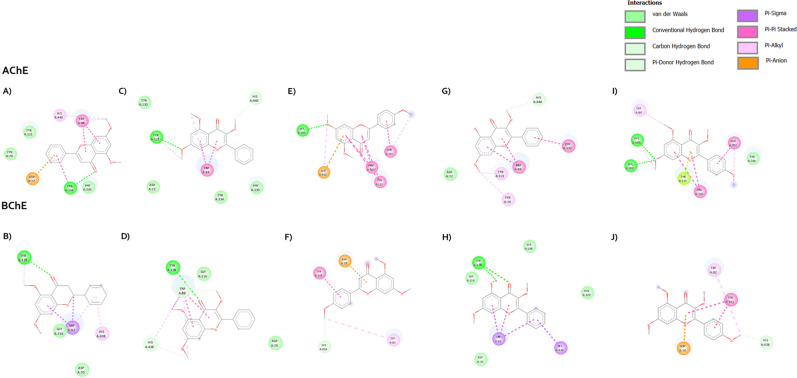
A visual representation of the molecular interactions between methoxyflavones F1-F5 and cholinesterases (AChE and BuChE). The figure depicts specific binding sites and types of interactions. F1 (A and B), F2 (C and D), F3 (E and F), F4 (G and H), and F5 (I and J).

Molecular docking is a computational technique which provides insights into molecular interactions between ligands and receptors. In this study, molecular docking was performed and revealed the binding patterns of MFs against AChE and BChE. The exploration of AChE interaction with flavonoids found that the core structure of flavonoids positioned itself at the anionic site (AS) and engaged in π–π interactions with Trp84 (Figs 9A and 10). The most potent MF or F3 established the hydrogen bond interactions between 7-OMe to Ser200, 4-pyranone to Tyr334, and 4′-OMe to Asp72. Additionally, its ring A formed a π-anion interaction with His440 (Fig 10E). Both F1 and F3 adopted similar poses, with the 4-pyranone moiety of F1 forming a hydrogen bond with Tyr334 (Fig 10A). In contrast, while the others assumed different poses, they were still situated within the middle gorge. F2, identified as the least active MF, has only hydrogen bond interaction between 7-Ome and Tyr121 (Fig 10C). Regarding the docking interactions of 7-OMe for F4 and F5, the results revealed that F4 demonstrated π-alkyl interactions with Tyr121 and Tyr70. On the other hand, F5 established hydrogen bonds with His440 and Ser200 (Fig 10G and 10I). Similar to their interaction with AChE, when MF docked with BChE, they positioned themselves over the middle gorge (Fig 9B). The core structure of flavonoids (ring A and C) engaged in a π–π interaction with Trp82, while their 4-pyranone moiety established a hydrogen bond with Tyr128 in the anionic site (AS). The ring B of F1 can interact with His438 of catalytic triad via π-alkyl interaction. The methoxy substitution on C-5 and C-7 had π-alkyl interaction with Tyr128, and van der Waals force with Asn83, respectively (Fig 10B). The methoxy group of F2 on C-5 and C-7 formed hydrophobic interactions with Trp82, and Ala328 (Fig 10D). For compound F4, the 7-OMe formed the π -alkyl with Pro84. Furthermore, there is a hydrogen bond interaction to the hydroxyl at C-5 which is found with Tyr128. Ring B of compound F4 established the π-sigma and π-alkyl with His438, Trp82, and Ala328 (Fig 10H). Apart from the others, the core structure of F3 and F5 is in the opposite orientation. Their ring B and C can form π–π interaction with Tyr332, and π-anion with Asp70, respectively. Their 4′-OMe formed the π-alkyl interactions with Trp82, Trp430, Tyr440, Ala328, and Met437. The methoxy at C-7 established the π-alkyl with Pro84 and Ile69 (Fig 10F and 10J). Interestingly, all methoxy substitutions of MF at C-3 did not form any interactions with BChE amino acid residues.

**Fig 11:**
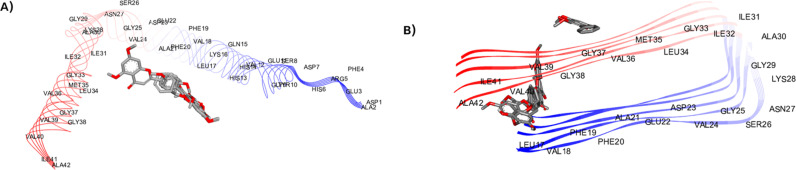
Binding mode of MFs at the binding site of Aβ monomer (A) and Aβ fibrils (B), as determined through molecular docking simulations.

**Fig 12:**
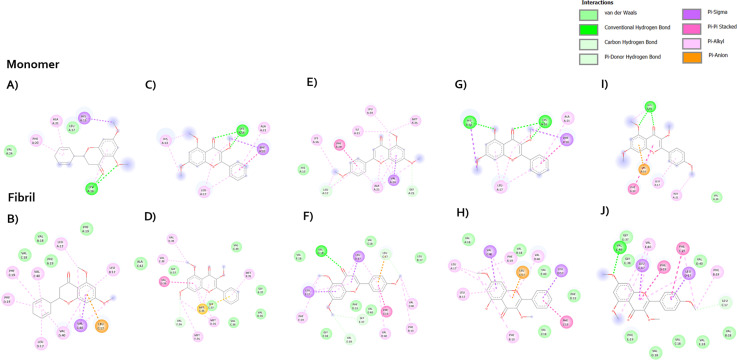
Molecular interactions of methoxyflavones F1-F5 with Aβ monomer and fibrils. The figure highlights key interactions and binding sites. F1 (A and B), F2 (C and D), F3 (E and F), F4 (G and H), and F5 (I and J).

Furthermore, the assembly of amyloid beta involves the intrastrand hydrogen bonding of the hydrophobic segments between Leu17-Ala21, and Ile31-Val36, which this process represents the nucleation of beta-sheet secondary structure [33]. Also, the binding of residues 17-21 of an incoming beta amyloid beta monomer could initially form the hydrophobic interaction to extend the fibril [34]. These amino acids, His13, Gln15, Phe19, Ile32, Leu34, Val36, Gly38 and Val40, are involved in the Aβ aggregation through the side chain packing. Next, the staggering of the individual beta-strands involves the side chain packing and form the hydrophobic interaction to connect two beta-strands [33]. The observation of MFs-Aβ monomer complex revealed that the core structure of flavonoid interacts with Leu17, Phe20, Ala21 of monomer. Their substitutions on C-3, C-5 and C-7 can form the hydrophobic, and hydrogen bond interactions with His13, and Lys16. These ligands are embedded in hydrophobic surface grooves parallel to the monomer (Figs 11A and 12). Aside from the others, the methoxy group of C-3 and C-7 on compound F5 did not establish the interactions with monomer which led to a decrease in inhibition activity (Fig 12I). The methoxy on C-4′ interacted with Lys16, Leu17, and Ala21. When the MFs docked with Aβ fibril, the flavonoid ring can be capable of entering into the Aβ fibril and form the hydrophobic interaction with Leu17, Phe19, Met35, Val36, and Val40 (Figs 11B and 12). These residues play a key role in the intermolecular contacts between β-strands. However, only F2 binds to the outside the groove which interacts with the surface of the fibrils. The binding interaction of F2 core structure still form the π -alkyl and π–π stacking interaction with Met35 and Val36, respectively. The methoxy at C-5 and C-7 can form the π -alkyl interaction with Met35 (Fig 12D). In the meantime, the substituents on C-3 and C-5 of F4 did not show any interactions with key amino acids, but methoxy group at C-7 form the π -alkyl interaction with Leu17 and Phe19 (Fig 12H). Considering the binding interaction of compound F1, F3 and F5 (Fig 12B, 12F, and 12J), the methoxy substituents at C-3 did not have the potential to form the interaction. The methoxy at C-5 and C-7 established hydrophobic interactions with Leu17, Val39, and Val40. Interestingly, the methoxy group at C-4′ can develop an effective interaction with Leu17, Phe19, and Val40.

#### Structure-activity relationship (SAR).

SAR studies elucidate the relationship between structural features of a compound and its biological activity. The active site of AChE is a catalytic triad (Ser200, His440, and Glu327), but three amino acids of catalytic triad are Ser198, His438, and Glu325 in BChE. This triad is responsible for the hydrolysis of ACh, cleaving it into acetate and choline. The AS consists of the negatively charged amino acid residues (Trp84, Tyr130, Tyr330, Phe331), which are important for the binding of quaternary ammonium ligands by π-cation interactions to make the appropriate orientation of ACh during catalytic process. Trp82 in BChE is conserved, however, Phe330 is replaced by Ala328, leading to the inhibitor affinities. In the acyl pocket, the amino acids in AChE and BChE are aromatic (Phe288 and Phe290) and aliphatic (Leu286 and Val288) amino acids, respectively. The acyl pocket of BChE has a larger size than AChE because of the smaller amino acids. Thus, these had influenced the substrate specificity. The phenylalanine of AChE can prevent the entry of the larger molecules to the catalytic site. The peripheral site (PAS) is located at the entrance of the active site cleft and can interact with Aβ, leading to accelerate amyloid aggregation. It consists of 5 amino acid residues (Tyr70, Asp72, Tyr121, Trp279 and Tyr334) that form cluster around the entrance of enzyme. However, Tyr70, Tyr121 and Trp279 in the PAS are not reserved for BChE [35].

For SAR study of MFs on AChE inhibition, the binding patterns of MFs revealed that the core structure of flavonoid lied on the AS. This may lead to the inhibitory effect of flavonoids on AChE activity. The most potent compound toward AChE is F3. It established the hydrogen bond interactions between 7-OMe to Ser200, and its ring A formed π-anion with His440. The serine residue in the active site plays a crucial role in the catalytic mechanism. It forms a covalent bond with the acetyl group of ACh, which is then cleaved by a water molecule that is activated by the histidine and glutamate residues in the triad. This might support the most inhibitory activity of it. In accordance with F3 and F1, the methoxy at C-4′ on ring B enhanced the inhibitory effect via forming hydrogen bond with Asp72. The addition of methoxy at C-4′ is confirmed that it is important for AChE inhibition in agreement with previous reported data [36]. This might be important for the interaction with PAS which increases the binding affinity of F3. Although F5 has the substitution of the methoxy at C-4′ on ring B but the inhibitory activity was not different compared to F2. The potency of F2, F4, and F5 significantly reduced when they are substituted by methoxy group at C-3. According to molecular docking, the steric hindrance might occur from the addition of methoxy group at C-3 because their binding poses are completely different from F1 and F3. These might suggest that the substitution of methoxy group at C-3 reduced the AChE inhibitory activity. Furthermore, our computational studies between BChE and MFs revealed that the flavonoid core structure located in AS is like AChE. However, F3 and F5 are in the opposite position, anchoring in PAS. This binding pose is possible to be a steric effect from the substitution of methoxy group at C-4′ position. The replacement of hydroxyl group at C-5 enhanced the BChE inhibition of compound F4 by increasing the binding affinity via hydrogen bonding. Furthermore, it might influence the ligand binding orientation which resulted in hydrophobic interaction between ring B and His438. It is interesting to note that the methoxy substituent at C-3 didn’t form the interaction with any amino acids. But it might cause some steric hindrance to affect the binding affinity with an enzyme, leading to reducing the inhibitory activity.

**Fig 13:**
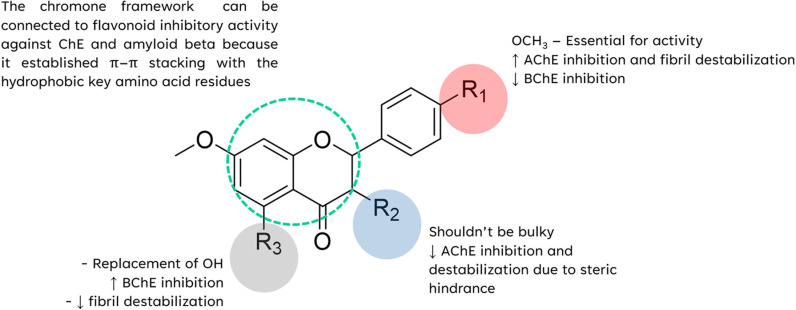
Structure-activity relationship of MFs on anti-AD effects.

In AD brain, there is a protein namely amyloid which can accumulate and become plaques. These amyloid plaques are thought to be toxic to the neurons, and the most toxic form is Aβ_1-42_. The complex of MFs and Aβ monomer was stabilized with Lys16, Leu17, Ala21, Val24 through the hydrophobic interactions. These interacting residues are present in the hydrophobic pockets and amyloidogenic regions of Aβ_42_ peptide and their interactions with MFs can prevent the aggregation of Aβ_42_ monomers to form toxic aggregates. The SAR analysis showed that the substitutions at C-5 and C-7 on MFs did not show any difference interactions. There is no different interaction when the methoxy group is replaced by the hydroxyl group at C-5. Increasing the number of substitutions seemed to correlate to higher activity. Thus, three substituents on ring A and C might be proper to avoid steric clashes when ligands bind to monomers. Furthermore, the substitution on ring B could increase the chance to form more favorable interactions. Next, our study used the five chains of Aβ_42_ as the Aβ fibril structure. The binding analysis revealed that MFs inserted into a groove and did not parallel with the fibril axis, supporting the destabilization of MFs on Aβ aggregates. However, F2 binds to the outside the groove which interacts with the surface of the fibrils. This might lead to its low effect on destabilization process. According to the binding interaction, the methoxy at C-5 and C-7 can form the interaction with Leu17, Val36, Val39 which are the hydrophobic core residues, leading to the instability of the Aβ. There is no difference between the hydroxyl and methoxy group at C-5, which is similar to the inhibition of fibril formation. Furthermore, the methoxy substitution on C-4′ is necessary for the destabilization because Phe19 and Gly38 appeared to form a hydrophobic interaction pair, connecting the two interstrands. It is interesting to note that the methoxy at C-3 did not have interactions with any amino acids. Compared to F3, the presence of methoxy C-3 might provide the steric effect which reduced the activity of F5. This indicates that the substitution at C-4′ might be important for the Aβ destabilization. SAR summary profile is summarized in Fig 13.

### In vivo evaluation of KP extract in reversing scopolamine-induced cognitive impairment in mice

#### Locomotion activity.

**Fig 14:**
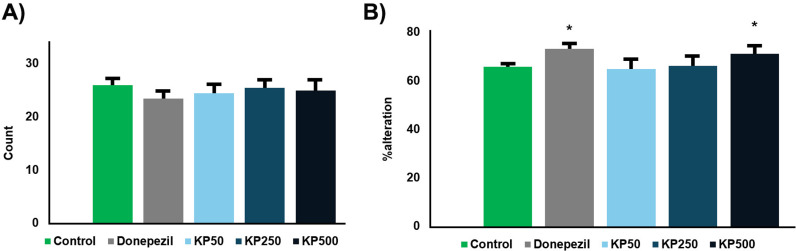
Effect of KP Extract (50, 250, and 500 mg/kg/day) on Y-Maze Behavior. (A) Locomotor Activity: The total number of arm entries in the Y-maze was measured to assess locomotor activity. (B) Spontaneous Alternation: The percentage of consecutive visits to all three arms was calculated to evaluate spontaneous alternation behavior. Data are presented as mean ±  standard error of the mean (SEM). Statistical significance was determined using a one-way ANOVA followed by a post-hoc Tukey’s HSD test. * p <  0.05, **p <  0.01 compared to the scopolamine-treated group. Donepezil at 3 mg/kg/day was used as a positive control.

The locomotor test is an essential component in mice model studies for several reasons. Primarily, it assesses the general activity levels and motor function of the mice, which is crucial for determining whether any observed cognitive impairments or improvements are due to changes in locomotor activity rather than specific effects on learning and memory. Additionally, it helps identify potential side effects of tested compounds on motor abilities, which could confound the results of cognitive tests [37]. In this study, mice were evaluated for their locomotor activity in Y-maze to assess whether our treatment did not have any effects on their movements. The results revealed that there is no significant difference in all treatment groups (Fig 14A). Moreover, the percentage of spontaneous alteration has been employed to investigate the ability to enhance memory. Donepezil and KP500 showed the higher percentage of spontaneous alteration than control group (*p* < 0.05) (Fig 14B). This suggested that the donepezil and KP500 might improve the memory recognition in healthy mice.

#### Modified Y-maze.

**Fig 15:**
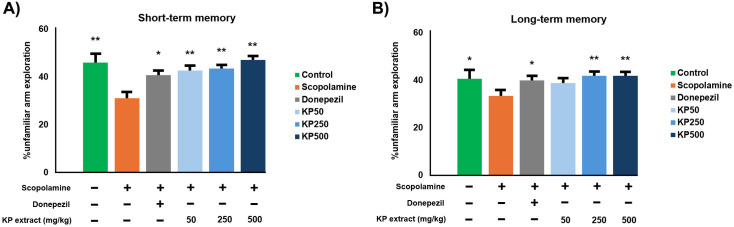
Reversal of scopolamine-induced memory impairment by KP extract in the modified Y-maze. (A) Short-Term Memory: Spatial memory was assessed 30 min after training. (B) Long-Term Memory: Spatial memory was evaluated 24 hours after training. Data are presented as mean ±  standard error of the mean (SEM. Statistical significance was determined using a one-way ANOVA followed by a post-hoc Tukey’s HSD test. * p <  0.05, **p <  0.01 compared to the scopolamine-treated group. Donepezil at 3 mg/kg/day was used as a positive control.

To investigate the impact of KP extract on spatial memory impairment, we conducted modified Y-maze tests in mice to evaluate memory retention by assessing delayed spontaneous alteration [38] Scopolamine served as a positive control, inducing spatial memory deficits in the mice. Compared to the control group, the scopolamine-treated group exhibited significantly reduced spatial recognition memory (*p* < 0.01). Interestingly, KP extracts demonstrated a dose-dependent increase in unfamiliar arm exploration compared to the scopolamine group. This suggests that pre-treatment with KP extracts ameliorated the impairment of both short- and long-term spatial memory (Fig 15A and 15B). However, it is important to note that the KP50 dose did not fully reverse the spatial long-term memory deficits induced by scopolamine in the modified Y-maze test (Fig 15B).

#### Novel objective recognition.

**Fig 16:**
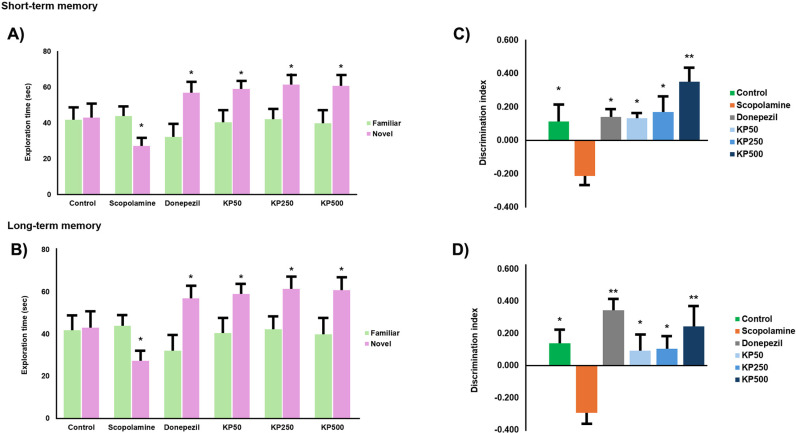
The impact of KP extract on memory impairment induced by scopolamine (1 mg/kg) in the novel object recognition test: (A) and (C) 5-min delay test (short term memory); (B) and (D) 24‐h delay test (long term memory). Data are presented as mean ±  SEM. * p <  0.05, **p <  0.01, between scopolamine group and treatment group. Donepezil at 3 mg/kg/day was used as standard reference.

The novel object recognition test was employed to assess changes in recognition memory, focusing on non-spatial memory within an open field. It determined the ability of mice to differentiate between familiar and novel objects based on their memory of previously encountered objects [39]. We observed memory impairment in short-and long-term memory as reflected by the significantly increased discrimination index of the novel object over the familiar object. The exploration time in short and long-term memory significantly decreased when compared to treatment group. Therefore, the administration of KP extract increased the exploration time which mice spent more time to discover novel object at all doses (Fig 16A and 16B). The group receiving donepezil also showed a significant increase in time spent with the novel object. Furthermore, the discrimination index was observed and there was a significant decrease of the discrimination index in scopolamine group compared to treatment group. Donepezil as the reference compound also increased this index of 0.141 ± 0.044 and 0.327 ± 0.072 for short and long-term memory, respectively (Fig 16C and 16D).

#### Morris water maze.

**Fig 17:**
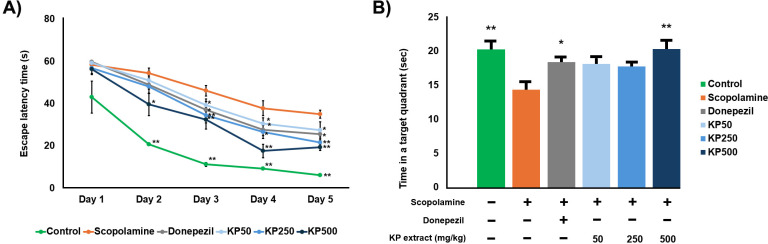
Effect of the KP extract on memory impairment induced by scopolamine in Morris water maze. (A) Escape latency in the acquisition phase and (B) time spent in the target quadrant during the probe test. Donepezil (3 mg/kg/day) was used as a reference standard. The data were shown as mean ±  SEM. *  *p* <  0.05, ** *p* <  0.01 compare with scopolamine-treated group.

The Morris water maze was utilized to assess the capacity for learning and retention of the location of a hidden platform within a spatial environment, evaluating alterations in both escape latency (time to find the platform) and the duration spent in the target quadrant. These measures respectively indicate enhanced learning efficiency and improved memory retention [40]. The effect of KP extract on spatial memory was expressed as the average latency of mice to reach the platform in the acquisition phase. The scopolamine group exhibited a significantly delayed mean escape latency time compared with the control group from day 2 to 5 in the acquisition phase (Fig 17A). Escape latencies in each group demonstrated a gradually decreasing profile. All KP concentrations showed significantly reduced escape latency compared to the scopolamine group on the day 3. The daily treatments could improve the spatial memory compared to the scopolamine group. The probe test was performed on the day 6 to check memory by measuring exploration time in the platform quadrant (Fig 17B). The scopolamine group showed a memory deficit and spent less time (14.37 ±  1.04) than the control group (20.27 ±  1.07) in the platform quadrant. However, only KP500 group spent more time exploring the platform (20.31 ± 1.25) than the scopolamine group. Therefore, we demonstrated that high KP dose could significantly reverse scopolamine-induced memory impairment (*p* < 0.01). Moreover, no significant differences were observed in mice among the control, KP500 and donepezil groups.

## Discussion

AD drug development is a global challenge, and the search for effective treatments remains ongoing. This research explored the promising potential of the KP and its constituents against the disease. MF is one type of flavonoid which is mostly found in KP rhizome, and exerts various beneficial pharmacological properties [41–43]. In this study, we evaluated the key biological activities linked to the AD pathological cascades including antioxidant capacity, cholinesterase inhibition, amyloid plaque reduction and neuroprotection. Additionally, we investigated the ability of KP to improve memory deficit in a mouse model. These findings could pave the way for developing novel therapeutic strategies aimed at slowing AD progression and protecting cognitive function. Our HPLC quantification of the KP extract identified F1 as a major compound, consistent with previous studies [42,44]. Therefore, F1 could serve as a promising quality control marker for KP raw material powder, addressing challenges in standardizing KP extracts for research and medicinal applications.

According to AD pathology, AChE is an enzyme responsible for breaking down the ACh neurotransmitter in the brain. ACh plays a crucial role in various cognitive functions such as learning and memory. The first-line therapeutic approach for alleviating the AD cognitive symptoms involves the use of AChE inhibitors (AChEIs) [27]. These inhibitors work by preventing the breakdown of ACh, thereby increasing its availability in the brain and potentially enhancing cognitive function. However, the effectiveness of AChEIs is often limited over time due to the decreased levels of AChE in severe AD compared to BChE [28]. Moreover, the increasing BChE levels in advanced AD raise concerns about the efficacy of AChE-selective inhibitors [45–47], as BChE serves as a compensatory enzyme in the progression of AD [29,30]. Therefore, dual inhibition of both AChE and BChE could present a promising strategy to alleviate the AD symptoms in both its mild and severe stages. Herein, we evaluated the efficacy of KP extract and MFs in cholinesterase inhibition, and the results demonstrated that both inhibited the activities of both enzymes. Therefore, mixed AChE/BChE inhibitors, such as MF and KP extracts, which exhibit balanced activity against both enzymes, could offer broader therapeutic potential for the AD treatment across both early and late stages. Further research is needed to optimize their potency and delivery for potential clinical applications.

Aβ accumulation plays an important role in AD, contributing to oxidative stress, synaptic dysfunction, and neuronal loss [48]. Failure to clear Aβ efficiently leads to the onset of AD. Therapeutic strategies thus focus on preventing Aβ aggregation, reducing its production, and promoting Aβ removal [49]. The potential of KP extract and its MFs in modulating Aβ_1–42_ aggregation and stability was investigated, revealing that both effectively hinder Aβ_1–42_ aggregation and destabilize existing fibrils. High levels of Aβ plaques can cause deleterious effects [46], with Aβ_1-42_ accumulation, particularly in cholinergic neurons of the brain, contributing to the neuronal damage observed in AD [46–48]. The SH-SY5Y cell line, known for its susceptibility to Aβ_1-42_ toxicity, is widely used to study neuroprotective effects [49–51]. KP extract and MFs protected SH-SY5Y cells from Aβ_1-42_-induced damage, suggesting their potential as neuroprotective agents in AD. To sum up, our compounds possess neuroprotective functions and can inhibit Aβ aggregation and promote its destabilization. These properties offer several beneficial effects as potential treatments in both early and late stages of AD, providing flexibility in treatment strategies. However, future research is crucial to fully elucidate the mechanism pathway for clearly unlocking their potential for AD treatment.

The presence of various components, including phenolic and flavonoid compounds, within the extract, likely contributes to its multi-faceted anti-AD effects through synergistic interactions [51–55]. Previous studies revealed that MFs are key chemical constituents in KP extract [42,50]. Our findings support this, demonstrating the potent anti-AD activity of MFs and suggesting they may be primary contributors to the extract’s efficacy in inhibiting relevant pathways. PCA analysis was used to explore relationships between variables and revealed that both anti-cholinesterase activity and Aβ modulation influenced MF clustering. Notably, F3 exhibited superior performance in these areas, including anti-Aβ aggregation and destabilization. These results suggest that F3 may be particularly effective in addressing the multifaceted nature of AD compared to other MFs. Our study highlights the potential of MFs, especially F3, as promising multi-target agents for AD treatment.

To further elucidate the mechanisms of action of MFs, computational techniques including molecular docking, and structure-activity relationship (SAR) studies were utilized. Flavonoids including MFs are natural compounds with potential against several AD targets [54,55]. Their core structure, consisting of two benzene rings linked by an oxygenated heterocyclic ring, may contribute to cholinesterase inhibition through non-polar interactions with key active-site residues. Prior studies suggested that specific hydroxyl groups and ring C unsaturation influence flavonoid anti-AChE activity [56,57]. However, BChE inhibition and Aβ modulation remained less understood. In this study, we revealed the SAR of MFs on cholinesterase inhibition and Aβ modulation. Our MFs contains hydrophobic groups which are likely to form non-polar interactions with the target. The results indicated that hydrophobic interactions with key active-site amino acids are crucial for both AChE and BChE inhibition. The potency of cholinesterase inhibitor relied on the specific functional groups and the strength of binding interactions. Notably, F3 displayed the highest AChE inhibitory activity, while F4 was the most potent against BChE. Furthermore, SAR analysis in the case of Aβ showed that the number of substituents on the MFs influenced its anti-aggregation activity. More substitutions generally enhanced binding affinity and stabilized the complex with Aβ_1–42_, thereby interfering with fibril formation. However, F5, with its higher number of substituents, exhibited lower activity, possibly due to steric hindrance that impeded interaction with Aβ_1–42_. For Aβ fibril destabilization, our results pointed to the importance of a methoxy substituent at C-4′ in ring B for increased potency. Conversely, a methoxy group at C-3 appeared to decrease the destabilizing effect due to steric hindrance. Therefore, molecular docking analysis confirmed that the C-4′ substitution plays a role in anti-AD functions of MFs, highlighting its potential relevance in guiding the design of flavonoid-based cholinesterase inhibitors and Aβ modulation for future drug development purposes.

Extensive research highlights the potential of KP extract, a traditional medicinal plant used for centuries, in combating various diseases [41]. KP extract exhibited various biological activities related to neuroprotective effects, with several studies reporting its anti-AD properties [16–18,58–61]. Thus, KP extract demonstrated protective potential against neurotoxicity, neuroinflammation, and neurogenesis decline, potentially delaying AD progression. However, there are limited studies in animal models related to memory and cognition. Therefore, the present study further investigated the potential of KP extract to ameliorate scopolamine-induced cognitive dysfunction in a mouse model.

KP extract has a high safety margin for daily use. The acute and chronic toxicity of KP were assessed, and it does not show any obvious symptoms of toxicity [41,62]. During our experiments, there were no significant differences in the body weight of mice between control and all treatment groups. We evaluated the effect of KP extract on memory function in amnesic mice using modified Y-maze, novel object recognition, and water maze tests. A day prior to testing, we investigated locomotor activity, and our KP extract showed no significant difference compared to control. When analyzing the percentage of spontaneous alternation, we found that KP at a dose of 500 mg/kg significantly increased spontaneous alternation. A high percentage of alternation is considered indicative of good spatial memory [63,64]. This suggests that KP might enhance memory recognition in healthy mice at this concentration.

Scopolamine is a muscarinic antagonist that inhibits cholinergic neuronal activity, leading to the impairment of both short-term and long-term memory [65,66]. It is commonly used to induce amnesia in animal models of memory loss. In the modified Y-maze test, scopolamine administration decreased novel arm visits compared to treatment group, while an increase in unfamiliar arm exploration indicated improved spatial working memory [37]. Our results showed that scopolamine-induced memory decline is reversed after KP treatment in the modified Y-maze test. In the novel object recognition test, the open field test is used to evaluate the exploration behavior of mice in response to a novel environment and assess non-spatial memory [37]. Scopolamine significantly impaired memory performance by decreasing exploration time spent on the new object and lowering the discrimination index, suggesting a decline in learning and recognition. However, KP extract increased both exploration time and the discrimination index, potentially indicating improvements in both short-term and long-term non-spatial memory. In the Morris water maze, a test of spatial learning and reference memory, scopolamine increased latency to find the platform in the acquisition phase, and decreased time spent in the platform quadrant during the probe test. Conversely, KP500 mice exhibited enhanced search specificity of the platform location and spent more time in the platform quadrant compared to the scopolamine group, demonstrating improved learning about the platform’s location. Our findings across various behavioral tests indicated that KP extract has the potential to reverse scopolamine-induced memory impairments, improving both spatial and non-spatial memory. These results showed the therapeutic potential of KP extract for cognitive impairments.

KP demonstrated a multifaceted mechanism of action, including the modulation of cholinesterase activity, Aβ formation and destabilization, and anti-inflammatory effects [17]. This contrasts with conventional drugs like donepezil and rivastigmine, which primarily act as AChEIs to increase ACh levels, or memantine, which protects against excitotoxicity by modulating NMDA receptor activity. Unlike these conventional drugs that typically target a single target, KP extract possessed multi-target effects that can address multiple aspects of AD pathology. Other herbal extracts, such as Ginkgo biloba, have shown cognitive benefits in some studies, but its benefits have been discussed controversially, with varying results across different studies and populations [67–69]. This underscores the need to explore novel herbal extracts with potentially greater efficacy for AD treatment. Moreover, KP extract has been evaluated in several clinical trials and has demonstrated a favorable safety profile [70–72]. With minimal reported side effects compared to conventional AD drugs, KP extract could be a promising candidate as a safer alternative or adjunctive treatment for AD. While other functional foods provide anti-oxidant and neuroprotective benefits [73], such as curcumin, a polyphenol known for its potent antioxidant and anti-inflammatory effects, which has shown improvement in cognitive decline in animal studies. However, its clinical effectiveness remains inconclusive, and it is associated with gastrointestinal adverse events [74]. In contrast, the polymethoxyflavones purified from KP extract showed no clinically adverse effects in the studies [75,76]. This suggested the safety of combining these polymethoxyflavones, which might be useful for our future study in evaluating their potential synergistic effects.

## Conclusion

In this study, we explored the potential of KP and MFs as a medication for AD treatment. We identified the MF profiles using HPLC and demonstrated their multi-action effects on AD pathogenesis, including anti-cholinesterase activities, inhibition of Aβ aggregation, destabilization of Aβ fibrils, and neuroprotection against Aβ-induced cell damage. In silico simulations suggested that methoxy substitution at C-4′ in ring B is crucial for interaction with targeted proteins. Among the five MFs studied, F3 showed the most promising profile, indicating its potential as a lead compound for further research. These findings suggested the importance of quality control development for KP. Furthermore, our studies indicated that KP has an ability to inhibit cholinesterase activity and Aβ aggregation as well as promote Aβ destabilization, which could be responsible for its memory enhancement effects and modifying disease progression. KP extract can also reverse scopolamine-induced memory decline and enhance both spatial and non-spatial memory. Although substantial preclinical evidence supports the use of KP extract in AD models, further clinical research is necessary to confirm its efficacy and safety in humans and to explore the complete neuroprotective mechanisms of KP.

## Supporting information

S1 Table In vitro biological activities related to AD. (PDF)

S1 Fig Cytotoxicity evaluation of MFs and KP extract. (PDF)

S2 Fig Neuroprotection evaluation. (PDF)

S3 Fig Effect of KP extract on Y-Maze behavior. (PDF)

S4 Fig The modified Y maze test. (PDF)

S5 Fig The novel object recognition test. (PDF)

S6 Fig The Morris water maze test. (PDF)
