# Supplementary material for: Kaempferia parviflora extract and its methoxyflavones as potential anti-Alzheimer assessing in vitro, integrated computational approach, and in vivo impact on behaviour in scopolamine-induced amnesic mice
Source: PLoS One. 2025 Mar 10;20(3):e0316888. doi: 10.1371/journal.pone.0316888 (PMC11892870; doi:10.1371/journal.pone.0316888)
Supplement: S1 Table — (PDF) [file pone.0316888.s001.pdf]

**Table 1. In vitro biological activities related to Alzheimer's pathology (IC50) of MFs and KP extract. Data are represented as mean ± SD (n = 3).**

**Acetylcholinesterase (AChE) inhibition**

| Sample | AChE inhibition ($IC_{50}$) | | | | |
|---|---|---|---|---|---|
| | **1** | **2** | **3** | **Average** | **SD** |
| KP extract (µg/mL) | 100.13 | 102.06 | 99.63 | 100.61 | 1.29 |
| F1 (µM) | 181.80 | 183.67 | 178.42 | 181.30 | 2.66 |
| F2 (µM) | 239.45 | 234.89 | 237.13 | 237.15 | 2.28 |
| F3 (µM) | 107.07 | 104.81 | 106.12 | 106.00 | 1.14 |
| F4 (µM) | 219.05 | 218.16 | 215.51 | 217.57 | 1.84 |
| F5 (µM) | 209.92 | 208.28 | 209.06 | 209.08 | 0.82 |
| Tacrine | 0.22 | 0.23 | 0.22 | 0.22 | 0.01 |
| Donepezil | 0.08 | 0.08 | 0.07 | 0.07 | 0.00 |

**Butyrylcholinesterase (BChE) inhibition**

| Sample | BChE inhibition ($IC_{50}$) | | | | |
|---|---|---|---|---|---|
| | **1** | **2** | **3** | **Average** | **SD** |
| KP extract (µg/mL) | 21.73 | 24.00 | 23.23 | 22.99 | 1.15 |
| F1 (µM) | 92.10 | 92.92 | 91.55 | 92.19 | 0.69 |
| F2 (µM) | 239.52 | 234.09 | 225.83 | 233.15 | 6.89 |
| F3 (µM) | 158.72 | 146.71 | 150.52 | 151.98 | 6.14 |
| F4 (µM) | 36.86 | 32.12 | 31.37 | 33.45 | 2.97 |
| F5 (µM) | 158.69 | 168.80 | 165.27 | 164.25 | 5.13 |
| Tacrine | 0.01 | 0.01 | 0.01 | 0.01 | 0.00 |
| Donepezil | 9.84 | 9.58 | 9.13 | 9.52 | 0.36 |

### Inhibition of amyloid beta (Aβ) formation

| Sample | $IC_{50}$ | | | | |
|---|---|---|---|---|---|
| | 1 | 2 | 3 | Average | SD |
| KP extract (µg/mL) | 195.38 | 199.50 | 201.92 | 198.93 | 3.31 |
| F1 (µM) | 106.50 | 103.82 | 105.16 | 105.16 | 1.34 |
| F2 (µM) | 90.31 | 89.10 | 81.97 | 87.13 | 4.51 |
| F3 (µM) | 86.46 | 91.99 | 95.75 | 91.40 | 4.67 |
| F4 (µM) | 99.80 | 95.34 | 96.15 | 97.10 | 2.38 |
| F5 (µM) | 139.91 | 132.77 | 130.10 | 134.26 | 5.08 |
| Curcumin (µM) | 6.97 | 7.60 | 7.49 | 7.36 | 0.34 |

### Amyloid beta (Aβ) destabilization

| Sample | $IC_{50}$ | | | | |
|---|---|---|---|---|---|
| | 1 | 2 | 3 | Average | SD |
| KP extract (µg/mL) | 4.22 | 3.99 | 4.19 | 4.13 | 0.12 |
| F1 (µM) | 80.89 | 70.97 | 75.93 | 75.93 | 4.96 |
| F2 (µM) | 82.20 | 83.86 | 85.53 | 83.86 | 1.67 |
| F3 (µM) | 39.37 | 38.91 | 40.13 | 39.47 | 0.61 |
| F4 (µM) | 65.46 | 64.43 | 61.90 | 63.93 | 1.83 |
| F5 (µM) | 52.96 | 47.66 | 53.17 | 51.26 | 3.13 |
| Curcumin (µM) | 7.03 | 6.29 | 6.65 | 6.66 | 0.37 |

### Antioxidant activity using DPPH and ABTS assay

| Sample | Assay | $IC_{50}$ (µg/mL) | | | | |
|---|---|---|---|---|---|---|
| | | 1 | 2 | 3 | Average | SD |
| **KP extract** | **DPPH** | 555.60 | 550.03 | 551.45 | 552.36 | 2.89 |
| | **ABTS** | 308.00 | 309.32 | 302.87 | 306.73 | 3.41 |
| **Trolox (µM)** | **DPPH** | 24.35 | 24.46 | 25.07 | 24.63 | 0.39 |
| | **ABTS** | 77.38 | 80.36 | 86.54 | 81.43 | 4.67 |
