# Supplementary material for: Kaempferia parviflora extract and its methoxyflavones as potential anti-Alzheimer assessing in vitro, integrated computational approach, and in vivo impact on behaviour in scopolamine-induced amnesic mice
Source: PLoS One. 2025 Mar 10;20(3):e0316888. doi: 10.1371/journal.pone.0316888 (PMC11892870; doi:10.1371/journal.pone.0316888)
Supplement: S1 Fig — (PDF) [file pone.0316888.s002.pdf]

Fig 6. Cytotoxicity evaluation of MFs and KP extract.

Sample	Concentration (μM)	%Cell viability						
		1	2	3	4	5	Average	SD
Control	0	94.92	106.73	99.24	103.30	101.27	101.09	4.42
F1	0.1	107.88	103.67	103.67	103.31	100.66	103.84	2.59
	1	108.84	103.31	103.79	98.38	104.27	103.72	3.72
	10	105.23	105.83	102.22	96.69	106.92	103.38	4.12
	100	101.98	105.59	102.35	101.98	102.71	102.92	1.52
F2	0.1	103.19	107.76	105.47	96.45	101.02	102.78	4.34
	1	103.07	103.31	101.38	97.53	102.22	101.50	2.34
	10	103.43	103.31	103.31	96.45	105.23	102.35	3.39
	100	95.49	90.20	94.17	95.85	94.53	94.05	2.26
F3	0.1	103.43	98.48	103.05	94.54	104.70	100.84	4.23
	1	105.20	93.02	102.28	101.90	105.84	101.65	5.13
	10	103.30	101.40	103.43	100.63	102.28	102.21	1.21
	100	95.69	95.56	94.42	97.08	94.92	95.53	1.00
F4	0.1	106.22	95.69	103.17	102.41	102.92	102.08	3.87
	1	102.28	102.79	100.51	102.66	105.71	102.79	1.87
	10	99.49	97.08	106.47	102.03	107.49	102.51	4.45
	100	80.96	77.79	80.20	83.12	80.20	80.46	1.91
F5	0.1	102.28	96.96	104.41	93.77	102.13	99.91	4.39
	1	98.02	100.61	105.17	102.28	93.16	99.85	4.55
	10	103.95	98.78	105.17	93.16	93.16	98.84	5.72
	100	94.83	102.58	99.85	106.08	107.60	102.19	5.10
KP	0.1	95.90	96.05	101.06	102.89	96.35	98.45	3.29
	1	85.26	94.98	103.80	100.30	94.53	95.78	7.03
	10	90.58	105.02	95.59	95.74	108.05	99.00	7.27
	100	62.31	62.77	75.08	78.57	77.81	71.31	8.11
