# Supplementary material for: Kaempferia parviflora extract and its methoxyflavones as potential anti-Alzheimer assessing in vitro, integrated computational approach, and in vivo impact on behaviour in scopolamine-induced amnesic mice
Source: PLoS One. 2025 Mar 10;20(3):e0316888. doi: 10.1371/journal.pone.0316888 (PMC11892870; doi:10.1371/journal.pone.0316888)
Supplement: S2 Fig — (PDF) [file pone.0316888.s003.pdf]

Fig 7. Neuroprotection evaluation against Aβ-induced cell damage

Sample	Concentration (μM)	%Cell viability						
		1	2	3	4	5	Average	SD
Control	0	101.33	97.71	100.82	99.82	100.32	100.00	1.40
Amyloid beta	25	62.42	69.66	62.12	68.46	68.56	66.24	3.66
Curcumin	10	83.33	84.64	71.87	73.18	76.00	77.80	5.85
F1	0.1	84.74	79.71	83.63	79.92	86.15	82.83	2.89
	1	82.23	88.06	83.53	90.07	88.76	86.53	3.44
	10	83.74	77.60	86.05	84.54	80.32	82.45	3.43
	100	82.93	72.78	82.73	87.45	74.39	80.06	6.23
F2	0.1	81.72	80.82	83.23	82.33	78.11	81.24	1.96
	1	85.04	78.01	88.16	81.02	85.95	83.63	4.07
	10	94.49	87.56	85.24	94.39	87.86	89.91	4.26
	100							
F3	0.1	79.51	77.10	78.31	69.56	75.19	75.93	3.90
	1	80.62	74.69	76.00	74.29	80.72	77.26	3.17
	10	74.79	82.03	77.70	77.70	81.72	78.79	3.06
	100	75.19	74.69	74.39	68.36	70.87	72.70	2.97
F4	0.1	79.71	79.92	79.31	78.41	81.22	79.71	1.02
	1	80.52	82.63	77.50	76.10	82.03	79.75	2.85
	10	79.51	82.43	84.54	82.53	80.42	81.89	1.97
	100							
F5	0.1	70.77	67.35	68.36	68.86	71.17	69.30	1.62
	1	70.77	72.38	70.27	67.15	68.05	69.72	2.11
	10	63.63	72.78	65.54	71.57	71.07	68.92	4.06
	100	68.25	66.65	65.54	68.76	70.37	67.91	1.88
KP	0.1	73.68	71.77	69.76	70.27	73.08	71.71	1.71
	1	76.80	79.21	69.56	73.58	75.09	74.85	3.62
	10	73.38	76.40	71.57	72.48	74.29	73.62	1.85
	100							
