# Supplementary material for: Kaempferia parviflora extract and its methoxyflavones as potential anti-Alzheimer assessing in vitro, integrated computational approach, and in vivo impact on behaviour in scopolamine-induced amnesic mice
Source: PLoS One. 2025 Mar 10;20(3):e0316888. doi: 10.1371/journal.pone.0316888 (PMC11892870; doi:10.1371/journal.pone.0316888)
Supplement: S3 Fig — (PDF) [file pone.0316888.s004.pdf]

Fig 14. Effect of KP Extract (50, 250, and 500 mg/kg/day) on Y-Maze Behavior. (A) Locomotor Activity: The total number of arm entries in the Y-maze was measured to assess locomotor activity. (B) Spontaneous Alternation: The percentage of consecutive visits to all three arms was calculated to evaluate spontaneous alternation behavior.

A: Locomotor

	**Average Count**				
	**Control**	**Donepezil**	**KP50**	**KP250**	**KP500**
	29	26	30	31	19
	28	29	23	28	26
	28	21	15	23	25
	28	20	25	30	19
	27	22	27	18	21
	21	27	28	26	35
	22	20	22	23	29
**Average**	**26.14**	**23.57**	**24.29**	**25.57**	**24.86**
**SEM**	**1.22**	**1.39**	**1.87**	**1.73**	**2.21**

B: Percentage of alteration

	**Average of %alteration**				
	**Control**	**Donepezil**	**KP50**	**KP250**	**KP500**
	65.00	75.86	63.89	83.87	65.38
	63.33	69.44	73.33	57.14	72.00
	65.52	75.00	78.57	66.67	78.95
	62.96	72.73	64.00	61.11	76.00
	57.78	77.78	81.48	60.87	71.43
	60.71	80.00	50.00	80.00	74.29
	64.29	68.18	72.73	69.23	79.31
**Average**	**62.80**	**74.14**	**69.14**	**68.41**	**73.91**
**SEM**	**1.03**	**1.62**	**4.06**	**3.82**	**1.84**
