# Supplementary material for: Kaempferia parviflora extract and its methoxyflavones as potential anti-Alzheimer assessing in vitro, integrated computational approach, and in vivo impact on behaviour in scopolamine-induced amnesic mice
Source: PLoS One. 2025 Mar 10;20(3):e0316888. doi: 10.1371/journal.pone.0316888 (PMC11892870; doi:10.1371/journal.pone.0316888)
Supplement: S4 Fig — (PDF) [file pone.0316888.s005.pdf]

Fig 15. Reversal of scopolamine-induced memory impairment by KP extract in the modified Y-maze. (A) Short-Term Memory: Spatial memory was assessed 30 min after training. (B) Long-Term Memory: Spatial memory was evaluated 24 hours after training. Data are presented as mean ± standard error of the mean (SEM. Statistical significance was determined using a one-way ANOVA followed by a post-hoc Tukey's HSD test. *p < 0.05, **p < 0.01 compared to the scopolamine-treated group. Donepezil at 3 mg/kg/day was used as a positive control.

(A) Modified Y-maze (Short-term memory; Percentage of unfamiliar arm exploration)

	Average of %unfamiliar arm exploration					
	**Control**	**Scopolamine**	**Donepezil**	**KP50**	**KP250**	**KP500**
	55.56	38.89	39.13	36.36	41.18	50.00
	42.86	35.71	38.46	44.44	50.00	43.75
	35.00	34.62	37.50	44.44	46.15	46.67
	42.86	27.78	47.37	50.00	42.86	52.38
	60.00	30.43	37.50	41.18	45.45	50.00
	42.86	22.22	47.50	37.50	40.91	44.44
	43.75	28.00	38.10	45.45	38.46	42.86
**Average**	**46.13**	**31.09**	**40.79**	**42.77**	**43.57**	**47.16**
**SEM**	**3.25**	**2.15**	**1.73**	**1.80**	**1.47**	**1.39**

(B) Modified Y-maze (Long-term memory; Percentage of unfamiliar arm exploration)

	Average of %unfamiliar arm exploration					
	**Control**	**Scopolamine**	**Donepezil**	**KP50**	**KP250**	**KP500**
	42.11	32.26	41.67	37.50	36.84	40.00
	42.86	34.78	38.89	35.71	50.00	41.18
	45.45	35.71	41.67	43.48	40.00	44.44
	41.38	30.00	46.67	45.45	42.86	42.11
	33.33	31.82	37.50	36.36	35.71	45.45
	44.44	35.71	36.84	33.33	42.11	42.86
	35.00	33.33	36.36	40.00	45.45	37.50
**Average**	**40.65**	**33.37**	**39.94**	**38.83**	**41.85**	**41.93**
**SEM**	**1.76**	**0.82**	**1.39**	**1.65**	**1.87**	**1.02**
