# Supplementary material for: Kaempferia parviflora extract and its methoxyflavones as potential anti-Alzheimer assessing in vitro, integrated computational approach, and in vivo impact on behaviour in scopolamine-induced amnesic mice
Source: PLoS One. 2025 Mar 10;20(3):e0316888. doi: 10.1371/journal.pone.0316888 (PMC11892870; doi:10.1371/journal.pone.0316888)
Supplement: S5 Fig — (PDF) [file pone.0316888.s006.pdf]

Fig 16. The impact of KP extract on memory impairment induced by scopolamine (1 mg/kg) in the novel object recognition test: (A) and (C) 5-min delay test (short term memory); (B) and (D) 24-h delay test (long term memory). Data are presented as mean ± SEM. *$p < 0.05$, **$p < 0.01$, between scopolamine group and treatment group. Donepezil at 3 mg/kg/day was used as standard reference.

(A) Novel object recognition (Short-term memory; Time spent exploring the familiar object)

	Average time spent (sec)					
	**Control**	**Scopolamine**	**Donepezil**	**KP50**	**KP250**	**KP500**
	66.38	62.12	53.71	52.17	54.38	30.97
	47.44	79.70	57.86	47.23	40.06	39.59
	42.78	55.96	54.10	44.46	50.68	43.65
	32.76	35.66	42.16	45.92	58.11	34.70
	55.25	69.38	57.20	56.45	66.52	54.82
	96.40	79.48	46.16	47.09	33.32	80.90
	54.50	61.48	58.80	70.39	53.70	67.29
**Average**	**56.50**	**63.40**	**52.86**	**51.96**	**50.97**	**50.27**
**SEM**	**7.76**	**5.75**	**2.39**	**3.45**	**4.20**	**6.94**

(A) Novel object recognition (Short-term memory; Time spent exploring the novel object)

	Average time spent (sec)					
	**Control**	**Scopolamine**	**Donepezil**	**KP50**	**KP250**	**KP500**
	74.78	40.43	66.34	64.15	79.73	85.78
	54.25	36.30	64.08	57.67	67.13	93.51
	39.51	40.08	66.20	57.78	59.60	81.29
	24.39	36.19	64.16	84.03	77.72	103.90
	55.20	61.90	73.07	60.10	58.50	60.55
	44.18	58.51	46.76	71.81	60.40	64.27
	84.48	54.18	69.59	67.34	54.81	56.47
**Average**	**53.83**	**46.80**	**64.31**	**66.12**	**65.41**	**77.97**
**SEM**	**7.78**	**4.16**	**3.16**	**3.57**	**3.71**	**6.80**

(C) Novel object recognition (Short-term memory; Discrimination index)

	**Control**	**Scopolamine**	**Donepezil**	**KP50**	**KP250**	**KP500**
	0.06	-0.42	0.33	-0.03	0.06	0.41
	0.07	-0.24	0.07	0.10	0.33	0.30

	-0.04	-0.39	0.09	0.32	0.65	0.50
	-0.15	-0.17	0.27	0.12	0.21	0.68
	0.00	-0.06	0.01	0.15	-0.06	0.05
	0.63	-0.15	0.08	0.14	0.29	-0.02
	0.22	-0.06	0.15	0.13	0.01	0.43
**Average**	**0.113**	**-0.212**	**0.141**	**0.132**	**0.214**	**0.334**
**SEM**	**0.096**	**0.055**	**0.044**	**0.039**	**0.092**	**0.094**

(B) Novel object recognition (Long-term memory; Time spent exploring the familiar object)

	**Average time spent (sec)**					
	**Control**	**Scopolamine**	**Donepezil**	**KP50**	**KP250**	**KP500**
	74.27	29.53	24.83	59.44	59.03	34.42
	31.53	49.02	18.44	17.15	24.99	21.72
	46.96	30.94	71.24	16.49	40.44	20.11
	17.07	30.83	23.36	46.22	50.45	43.67
	43.86	53.50	39.73	51.80	56.06	77.00
	45.81	54.41	28.57	35.32	20.07	43.50
	33.07	58.58	18.58	56.11	44.07	38.52
**Average**	**41.79**	**43.83**	**32.11**	**40.36**	**42.16**	**39.85**
**SEM**	**6.72**	**4.85**	**7.07**	**6.75**	**5.64**	**7.17**

(B) Novel object recognition (Long-term memory; Time spent exploring the novel object)

	Average time spent (sec)					
	**Control**	**Scopolamine**	**Donepezil**	**KP50**	**KP250**	**KP500**
	21.51	35.10	51.61	66.63	59.97	49.30
	29.15	14.04	32.92	73.02	65.12	78.33
	61.63	15.47	70.88	50.88	54.96	78.50
	33.66	41.67	49.01	43.98	70.29	72.75
	39.96	39.85	60.94	47.38	56.90	46.20
	37.52	18.43	80.52	62.12	85.07	41.31
	77.42	25.89	51.99	68.93	37.48	59.00
**Average**	**42.98**	**27.21**	**56.84**	**58.99**	**61.40**	**60.77**
**SEM**	**7.42**	**4.42**	**5.89**	**4.34**	**5.54**	**5.96**

(D) Novel object recognition (Long-term memory; Discrimination index)

	**Control**	**Scopolamine**	**Donepezil**	**KP50**	**KP250**	**KP500**
	0.30	-0.33	0.59	0.06	0.01	0.18
	0.40	-0.55	0.38	0.51	0.45	0.57
	0.14	-0.18	0.28	0.16	-0.15	0.59
	-0.12	-0.15	0.00	-0.04	0.15	0.72
	-0.05	-0.13	0.35	-0.22	0.16	-0.19
	-0.10	-0.49	0.21	0.32	0.21	0.11
	0.40	-0.39	0.48	-0.14	-0.08	0.03
**Average**	**0.14**	**-0.32**	**0.33**	**0.09**	**0.11**	**0.29**
**SEM**	**0.09**	**0.06**	**0.07**	**0.10**	**0.08**	**0.13**
