# Supplementary material for: Kaempferia parviflora extract and its methoxyflavones as potential anti-Alzheimer assessing in vitro, integrated computational approach, and in vivo impact on behaviour in scopolamine-induced amnesic mice
Source: PLoS One. 2025 Mar 10;20(3):e0316888. doi: 10.1371/journal.pone.0316888 (PMC11892870; doi:10.1371/journal.pone.0316888)
Supplement: S6 Fig — (PDF) [file pone.0316888.s007.pdf]

Fig 17. Effect of the KP extract on memory impairment induced by scopolamine in Morris water maze. (A) Escape latency in the acquisition phase and (B) time spent in the target quadrant during the probe test. Donepezil (3 mg/kg/day) was used as a reference standard. The data were shown as mean ± SEM. * $p < 0.05$, ** $p < 0.01$ compare with scopolamine-treated group.

(A) Escape latency in Acquisition phase

Control					
	Time to find the platform, Escape latency (Second)				
	**Day 1**	**Day 2**	**Day 3**	**Day 4**	**Day 5**
	60	18.5	16.75	14.5	5.5
	49.25	20.25	10	11.5	4.25
	33.5	25.5	13	9.25	8.75
	34.25	20.75	9.5	9.5	6.5
	60	15.25	12	7.5	5.75
	34.25	19.25	7.75	7	5.5
	37	25.5	9.75	5.5	7.5
**Average**	**44.04**	**20.71**	**11.25**	**9.25**	**6.25**
**SEM**	**12.17**	**3.72**	**2.97**	**3.02**	**1.49**
**Scopolamine**					
	**Day 1**	**Day 2**	**Day 3**	**Day 4**	**Day 5**
	49	60	34.5	42.25	60
	60	56.5	44.75	38.5	51.75
	60	55	47.25	39.25	17
	60	49.25	40.25	30	41.25
	60	60	60	45.5	16.75
	60	48	52.25	43	30.75
	60	52.5	44.5	39	27.75
**Average**	**58.43**	**54.46**	**46.21**	**39.64**	**35.04**
**SEM**	**4.16**	**4.81**	**8.22**	**4.95**	**16.69**
**Donepezil**					
	**Day 1**	**Day 2**	**Day 3**	**Day 4**	**Day 5**
	60	47.5	58.25	60	46.5
	60	60	37.75	11.75	15.5
	60	47.75	46.25	20.25	7
	59	36	16	20.5	7
	60	48.25	44.25	25.25	45.25
	60	43.5	24.25	30.5	39.25
	60	60	32.5	25.75	18.5
**Average**	**59.86**	**49.00**	**37.04**	**27.71**	**25.57**

**SEM**	**0.38**	**8.62**	**14.22**	**15.40**	**17.58**
**KP50**					
	**Day 1**	**Day 2**	**Day 3**	**Day 4**	**Day 5**
	60	48.75	31.5	25	29.5
	60	52.75	46	34.75	27.75
	54.75	60	60	25	31.5
	60	37	44	38	42
	60	56.75	45	26.25	13
	60	42.5	25	30.5	22.5
	60	60	23.75	34.25	26.75
**Average**	**59.25**	**51.11**	**39.32**	**30.54**	**27.57**
**SEM**	**1.98**	**8.86**	**13.13**	**5.27**	**8.81**
**KP250**					
	**Day 1**	**Day 2**	**Day 3**	**Day 4**	**Day 5**
	60	60	57.5	49.25	21.75
	60	60	48	35.25	20.5
	48.25	55	28.75	10.75	12
	60	14.25	14	9.75	23
	60	45	35	26	32.25
	60	51.75	31	33.25	22
	49.75	50.75	27.75	22.25	20.25
**Average**	**56.86**	**48.11**	**34.57**	**26.64**	**21.68**
**SEM**	**5.38**	**15.85**	**14.27**	**14.06**	**5.93**
**KP500**					
	**Day 1**	**Day 2**	**Day 3**	**Day 4**	**Day 5**
	60	33.75	25.25	7	13
	35	48.5	24	22	25.75
	60	36.75	46	24.5	16.25
	60	57.5	38.75	8.75	14.75
	60	23.25	20.25	24.75	11.25
	60	39.5	38.5	17.5	27.5
	60	38.25	34.25	18.75	27
**Average**	**56.43**	**39.64**	**32.43**	**17.61**	**19.36**
**SEM**	**9.45**	**10.89**	**9.44**	**7.19**	**7.10**

(B) Time spent in the target quadrant during the probe test.

	**Time spent in the target quadrant (Second)**					
	**Control**	**Scopolamine**	**Donepezil**	**KP50**	**KP250**	**KP500**
	20.37	14.63	16.63	19.75	16.25	24.86
	18.30	12.91	16.35	15.00	16.18	14.57
	25.93	14.48	20.23	19.80	17.82	22.60
	18.50	19.93	20.04	18.58	18.73	20.52

	21.53	13.47	20.62	20.66	16.72	19.90
	17.55	11.01	16.61	15.49	20.17	18.15
	19.67	14.19	18.21	17.64	18.37	21.59
**Average**	**20.27**	**14.37**	**18.39**	**18.13**	**17.75**	**20.31**
**SEM**	**2.84**	**2.75**	**1.89**	**2.20**	**1.47**	**3.30**
